# Diagnosing Camurati–Engelmann disease—the age of whole-exome sequencing

**DOI:** 10.1093/rheumatology/keac670

**Published:** 2022-12-15

**Authors:** Deepak Nagra, Mark D Russell, Edward Alveyn, Surinder S Birring, David Elias, Sathiyaa Balachandran, James B Galloway

**Affiliations:** Centre for Rheumatic Diseases, King’s College London, London, UK; Centre for Rheumatic Diseases, King’s College London, London, UK; Centre for Rheumatic Diseases, King’s College London, London, UK; King’s College London, London, UK; King’s College Hospital NHS Foundation Trust, London, UK; Centre for Rheumatic Diseases, King’s College London, London, UK; Centre for Rheumatic Diseases, King’s College London, London, UK

Rheumatology key message

- Whole-exome sequencing provides an opportunity to rapidly diagnose rare diseases in routine clinical care.

Dear Editor, Camurati–Engelmann disease (MIM# 131300), an autosomal dominant disorder first described in 1920, is caused by mutations in the Transforming Growth Factor Beta 1 (*TGFB1*, MIM# 190180) gene, resulting in sclerotic bone disease [1,2]. The disease presents in childhood, most commonly with limb pain, fatigue and a waddling gait. The pattern of sclerosis is different to that seen in other conditions, such as malignancy or metastasis, as the sclerosis is not patchy but instead involves the entirety of the bone.

We present a 56-year-old male who has been under our care for multisystem sarcoidosis diagnosed 17 years ago, with disease distribution including cutaneous, pulmonary, bone and ocular lesions. The diagnosis was confirmed with histological evidence of granulomatous inflammation on lymph node biopsy. He was medicated with HCQ 200 mg once daily and MTX 15 mg once weekly, having previously received treatment with MMF and AZA. His immune modulation had been under review, with consideration of a TNFα inhibitor, due to persistent bone pain that was thought to be attributable to active sarcoidosis. The bone pain had first been noted 10 years previously, with progressive worsening subsequently. The original diagnosis of sarcoid bone involvement was based upon radiographs demonstrating multifocal sclerotic lesions in his pelvis, proximal and distal femora, and radius (Fig. 1).

**Figure 1.**
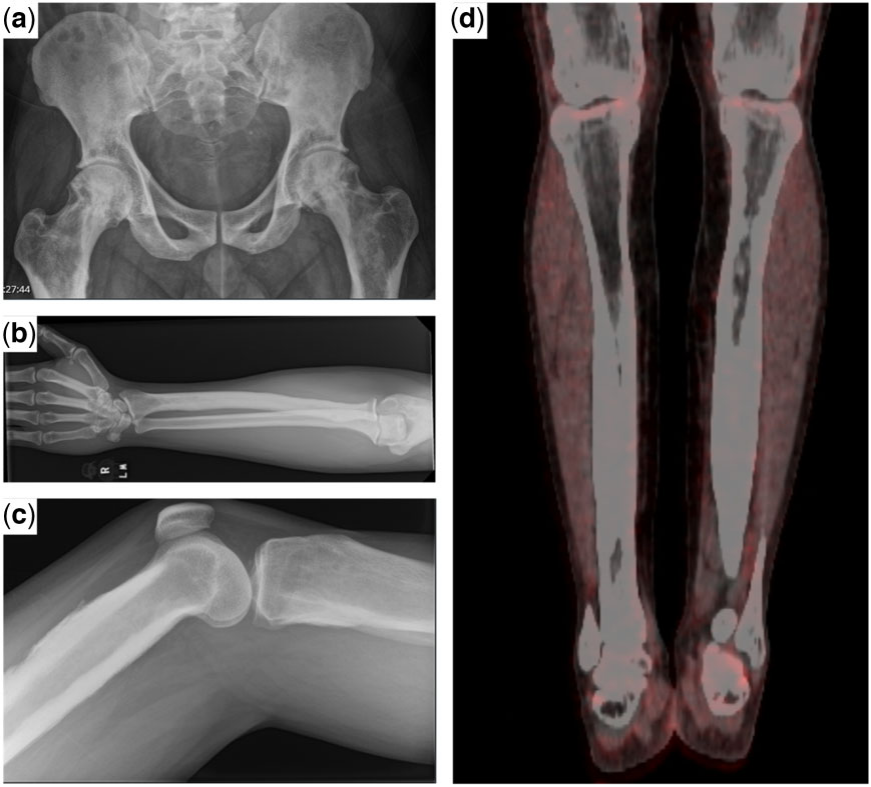
Imaging findings from the clinical case. Plain radiographs demonstrating osteosclerotic changes (panel a), with widespread diaphyseal dysplasia and cortical thickening in the long bones (panels b–c). Fluorodeoxyglucose (FDG)-PET images of the tibial bones demonstrate the symmetry of the changes, as well as the lack of FDG-avidity in the skeleton (panel d)

On revisiting the imaging, unusual features were noted. The bone lesions were accompanied by significant pain, which is an unusual feature of sarcoidosis: sarcoid bone lesions are more often asymptomatic [3]. Second, the distribution of the lesions was more extensive than that of typical sarcoid bone disease. Third, the skeletal changes showed no avidity on FDG-PET imaging (Fig. 1). Additionally, there was no evidence of active sarcoidosis in other organ systems.

Collectively, these findings prompted us to consider alternative explanations for the patient’s skeletal manifestations. Due to diagnostic uncertainty, and following multidisciplinary input, whole-exome sequencing was performed (platform: NONACUS Cell3 Target ExomeCG; sequenced on Illumina NovaSeq, with a Hg38 genome reference, without Sanger sequencing validation). This identified the presence of a pathogenic mutation in *TGFB1*, consistent with a diagnosis of Camurati–Engelmann disease. Our patient was heterozygous for the *TGFB1* p*.* C223W mutation: a novel mutation of *TGFB1*, affecting the latency-associated peptide of the protein and predicted to be pathogenic (ACMG class 5; predicted to be damaging in SIFT, Polyphen and MutationTaster pathogenicity algorithms; CADD scaled score: 27.9; transcript number: NM_000660). This mutation affects the same amino acid residue described in other published cases of Camurati–Engelmann disease [4]. No other relatives of the patient are known to be affected by Camurati–Engelmann disease.

Occam’s razor is a problem-solving principle that proposes ‘entities are not to be multiplied beyond necessity’ [5]. In hindsight, we attributed the skeletal lesions in this case to sarcoidosis, favouring the most parsimonious explanation despite the diagnostic incongruity. The advent of the NHS Genomic Service, launched in 2021, provides widespread access to whole-exome sequencing at no cost to NHS hospitals. This is perhaps an opportunity for clinicians to revisit some of our more puzzling cases.

For our patient, the discovery of an alternative cause for his skeletal lesions and pain has enabled us to cease our search for alternative immune modulation strategies. We have reassured him that his sarcoidosis is not active. Given the rarity of Camurati–Engelmann disease, there are no guidelines on optimal management. Case reports have described the use of steroids, bisphosphonates, denosumab and losartan, all with variable success [6]. Informed consent was obtained from the patient involved in this report. No further ethical approval was required.

## Data Availability

Not applicable.
